# Loss of CXCL12/CXCR4 signalling impacts several aspects of cardiovascular development but does not exacerbate Tbx1 haploinsufficiency

**DOI:** 10.1371/journal.pone.0207251

**Published:** 2018-11-08

**Authors:** Mahalia Page, Liam Ridge, Diana Gold Diaz, Tsolmon Tsogbayar, Peter J. Scambler, Sarah Ivins

**Affiliations:** Developmental Biology of Birth Defects, UCL Institute of Child Health, London, United Kingdom; Mayo Clinic, UNITED STATES

## Abstract

The CXCL12-CXCR4 pathway has crucial roles in stem cell homing and maintenance, neuronal guidance, cancer progression, inflammation, remote-conditioning, cell migration and development. Recently, work in chick suggested that signalling via CXCR4 in neural crest cells (NCCs) has a role in the 22q11.2 deletion syndrome (22q11.2DS), a disorder where haploinsufficiency of the transcription factor TBX1 is responsible for the major structural defects. We tested this idea in mouse models. Our analysis of genes with altered expression in *Tbx1* mutant mouse models showed down-regulation of *Cxcl12* in pharyngeal surface ectoderm and rostral mesoderm, both tissues with the potential to signal to migrating NCCs. Conditional mutagenesis of *Tbx1* in the pharyngeal surface ectoderm is associated with hypo/aplasia of the 4^th^ pharyngeal arch artery (PAA) and interruption of the aortic arch type B (IAA-B), the cardiovascular defect most typical of 22q11.2DS. We therefore analysed constitutive mouse mutants of the ligand (CXCL12) and receptor (CXCR4) components of the pathway, in addition to ectodermal conditionals of *Cxcl12* and NCC conditionals of *Cxcr4*. However, none of these typical 22q11.2DS features were detected in constitutively or conditionally mutant embryos. Instead, duplicated carotid arteries were observed, a phenotype recapitulated in *Tie-2Cre* (endothelial) conditional knock outs of *Cxcr4*. Previous studies have demonstrated genetic interaction between signalling pathways and *Tbx1* haploinsufficiency e.g. FGF, WNT, SMAD-dependent. We therefore tested for possible epistasis between *Tbx1* and the CXCL12 signalling axis by examining *Tbx1* and *Cxcl12* double heterozygotes as well as *Tbx1/Cxcl12/Cxcr4* triple heterozygotes, but failed to identify any exacerbation of the *Tbx1* haploinsufficient arch artery phenotype. We conclude that CXCL12 signalling via NCC/CXCR4 has no major role in the genesis of the *Tbx1* loss of function phenotype. Instead, the pathway has a distinct effect on remodelling of head vessels and interventricular septation mediated via CXCL12 signalling from the pharyngeal surface ectoderm and second heart field to endothelial cells.

## Introduction

*TBX1* haploinsufficiency is the major contributing factor in the development of congenital cardiovascular defects in the 22q11.2 deletion syndrome (22q11.2DS). Conditional mutagenesis experiments have determined the tissue specific and temporal requirements for this transcription factor in the mouse (reviewed in [1]). Notably, *Tbx1* is required in the pharyngeal surface ectoderm for the formation and remodelling of the embryonic pharyngeal arch artery (PAA) system into the great vessels.

Defects of the aortic arch and right subclavian artery (RSA) are prominent e.g. retro-oesophageal RSA. The defects observed in mice correlate well with abnormalities observed in human patients. In particular, interrupted artic arch type B (IAA-B), which represents a left 4^th^ pharyngeal arch artery (PAA) abnormality is quite specific for 22q11.2 deletion syndrome in that ~50% of patients presenting with this defect will test positive for the 22q11.2 deletion [2].

Furthermore, *Tbx1* nulls all have a common arterial trunk (CAT) [3, 4]; *Tbx1* is required in the second heart field for septation of the outflow tract, atrial and ventricular septation (specifically, closure of the membranous part of the septum) and correct alignment of the outflow tract with the ventricles.

We and others have identified defects of neural crest cell (NCC) patterning in *Tbx1* null and heterozygous embryos [5, 6]. Importantly, *Tbx1* is not expressed in NCCs and therefore such abnormalities of NCC patterning must be the result of defective signalling downstream of TBX1. We have attempted to identify pathways downstream of TBX1 by using a combination of dissection and FACS microarrays, comparing wild type with mutant tissue. For instance *Gbx2* expression in the pharyngeal surface ectoderm was shown to be dependent upon TBX1 and in turn *Slit2* signalling was affected when both *Tbx1* and *Gbx2* were deleted from the pharyngeal surface ectoderm [5]. Slit signalling is required for inter-ventricular septation [7]. However, such links cannot fully explain defects seen in *Tbx1*^*+/-*^ or *Tbx1*^*-/-*^ mouse models. We therefore interrogated our existing data set [8] and identified *Cxcl12* as encoding a candidate ligand for transmitting cell non-autonomous effects of *Tbx1*.

In non-mammalian models, CXCL12, acting via CXCR4 has been shown to be a major player in directing cell migration involving contact inhibition of locomotion (CIL) in NCCs [9, 10], and in the zebrafish lateral line it forms a self-directed gradient for cell migration [11–13]. In frog, disruption of CXCL12 signalling causes craniofacial defects [14]. In chick, the use of viral interference of CXCR4 activity in NCC (more specifically, the cardiac neural crest) led to various heart defects seen in 22q11.2DS [15]. The authors of this paper proposed that *Cxcl12/Cxcr4* are genetically downstream of *Tbx1* during pharyngeal NCC development and that reduction of CXCR4 signalling causes misrouting of pharyngeal NCCs in chick.

In this work we test the above hypothesis in mice by examination of *Cxcl12* expression in control and *Tbx1* mutant embryos, and phenotypic examination of several *Cxcl12/Cxcr4* mutants. We show that loss of *Tbx1* is associated with reduction in the level of *Cxcl12* expression within the pharyngeal surface ectoderm and craniofacial mesoderm. However, *Cxcl12* null mutants do not have the 4^th^ PAA defects (i.e. retro-oesophageal RSA and aortic arch interruptions) or great vessel septation defects typical of *Tbx1* haploinsufficiency. Instead we identified apparently duplicated carotids and abnormalities of the subclavian arteries. Internal cardiac defects did include ventricular septal defects (VSDs) and occasional outflow alignment abnormalities, as previously reported, but no outflow tract septation defects were observed. No significant genetic interaction between *Tbx1*, and *Cxcl12* /*Cxcr4* heterozygosity was detected in terms of 4^th^ PAA development. Conditional mutagenesis revealed requirements for expression of *Cxcl12* in pharyngeal surface ectoderm and second heart field mesoderm. Conditional mutation of *Cxcr4* in endothelial cells recapitulated the *Cxcr4* null phenotype whilst conditional mutation of *Cxcr4* in NCCs did not recapitulate the neck vessel defects, but partially penetrant VSD was detected. We conclude that, in mice, the CXCL12-CXCR4 pathway is at most a minor player downstream of *Tbx1* loss of function phenotypes.

## Results

### Tbx1 mutation is associated with reduced Cxcl12 expression in the pharyngeal apparatus

**Fig 1:**
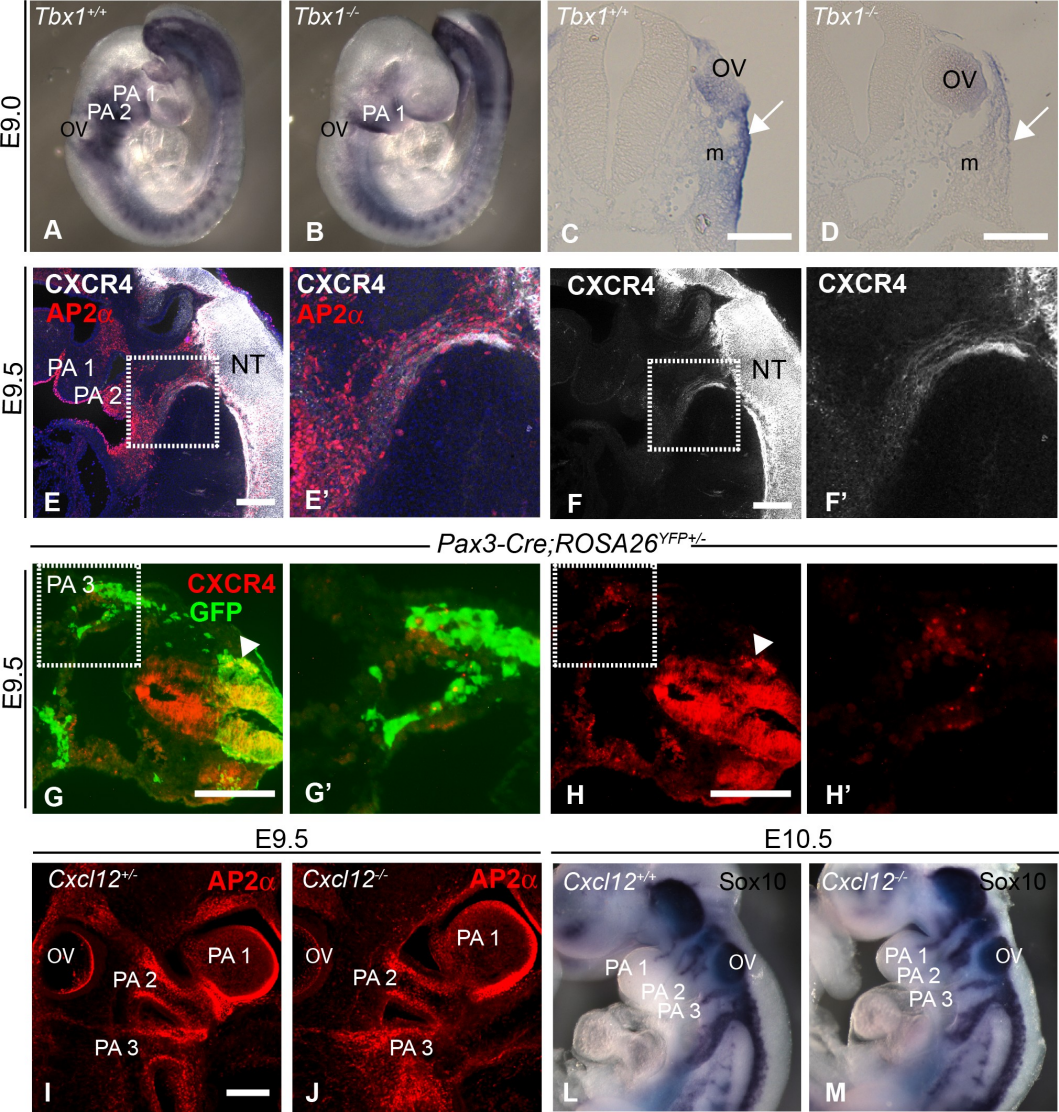
Expression of CXCR4/CXCL12 axis downstream of Tbx1 and in pharyngeal NCCs. **(A-D)** *Cxcl12* expression in Tbx1 mutant embryos at E9.0 (*in situ* hybridisations): wholemounts (A and B) and transverse sections at the level of PA2 (C and D). *Cxcl12* expression is reduced in the pharyngeal surface ectoderm (PSE, arrow) and underlying mesenchyme (m). **(E,F)** CXCR4 is expressed in migratory NCCs at E9.5. NCCs were detected in sagittal sections of wild type embryos using Ap2α antibody. CXCR4 is highly expressed in the neural tube (NT) and at a lower level in migrating NCCs (boxed regions in E and F, shown enlarged in E’ and F’). **(G, H)** CXCR4 is expressed in few lineage-traced NCCs in PA3 (Pax3-Cre;R26R^eYFP^ embryos) at E9.5 (boxed region in panels G and H, shown enlarged in G’ and H’) whilst high levels of CXCR4 can be observed in delaminating NCCs (arrowheads). CXCR4 expression was also observed in lineage-negative cells within PA3 (G(iii)). (**I- M)** NCC migration into the PAAs is normal in *Cxcl12* null embryos, as assessed by wholemount staining with Ap2α antibody (I, J, maximum z projections of confocal stacks) and *Sox10* expression (L, M, *in situ* hybridisations). OV, otic vesicle; NCCs,neural crest cells; PA3, pharyngeal arch 3;PSE, pharyngeal surface ectoderm. Scale bars represent 200μ in panels C-H and 100μ in panel I.

Our previous microarray analysis was aimed at identifying signalling processes downstream of TBX1 that might contribute to the *Tbx1* loss of function phenotype. *Cxcl12* was a product of this screen and its expression in *Tbx1*^*-/-*^ embryos was analysed by *in situ* hybridization. In addition to previously described expression in the outflow tract, we detected *Cxcl12* transcripts in the pharyngeal surface ectoderm of E9.0 embryos i.e. at the time when signalling from the pharyngeal surface ectoderm would be most likely to impact the underlying, migrating NCCs (Fig 1A and 1C). *Cxcl12* was expressed to a lesser extent in the rostral mesenchyme. Expression of *Cxcl12* was reduced, but not abolished, from these regions in *Tbx1*^*-/-*^ littermates (Fig 1C and 1D).

In agreement with previous observations in the chick [15, 16], CXCR4 is expressed in cardiac NCCs at E9.5 (Fig 1E–1F’), predominantly in delaminating cells and at a lower level in the migrating streams of NCCs (labelled with AP2α antibody). Interestingly, the majority of NCCs in the pharyngeal arches (PAs) themselves at E9.5 (as lineage-traced using Pax3-Cre crossed with ROSA26R^eYFP^ homozygotes) did not express CXCR4 (Fig 1G–1H’). In contrast to the chick experiments, NCC migration did not appear to be disrupted in *Cxcl12* null mouse embryos as shown by analysis of AP2α and *Sox10* expression (Fig 1I–1M). NCCs were localised normally within PAA1-3 in the nulls at E9.5 (compare Fig 1J to Fig 1I) and consistent with this, migrating streams of NCCs also appeared normal (Fig 1L and 1M). Thus there appear to be species differences pertaining to the role of CXCL12 signalling in NCC migration.

### Cardiovascular defects in CXCL12/CXCR4 Nulls are not those typical of 22q11.2DS or Tbx1 haploinsufficiency

Previous studies of *Cxcl12* and *Cxcr4* mutant mice have shown intracardiac defects, predominantly VSDs and abnormalities of the coronary arteries [17–21]. In this study we have focussed upon the cardiovascular defect most specifically associated with *Tbx1* haploinsufficiency–the morphogenesis of 4^th^ PAA derivatives. While IAA-B has been observed in several mouse models, examples of these defects in heterozygous as opposed to homozygous mutants are rare (*Chd7* heterozygosity being one example [22]). Defects of fourth arch structures are the most sensitive to reduced *Tbx1* dosage [23] and therefore, if CXCL12 is implicated downstream of TBX1 in arch morphogenesis, should be most sensitive to loss of CXCL12 signalling. We also specifically looked for retro-oesophageal RSA, a 4^th^ PAA defect more frequently observed in Tbx1 heterozygotes than IAA-B [5, 22, 24], and a common arterial trunk (CAT) which is seen in all *Tbx1* null embryos (and some 22q11.2DS patients) [3, 4], as well as other previously reported heart abnormalities. Defects are summarized in Table 1.

**Table 1:** Cardiovascular anomalies in *Cxcl12* and *Cxcr4* mutants at E15.5/E18.5.

Defect	Cxcl12^-/-^(n = 33)	Cxcr4^-/-^ (n = 5)	Cxcl12^+/-^ (n = 33)	Cxcl12 ^+/+^ (n = 26)
Aberrant LSA	28 *(84*.*8%)*	5 *(100%)*	0	0
Aberrant RSA	31 *(93*.*9%)*	5 *(100%)*	0	0
Duplicate LCC	24 *(72*.*7%)*	5 *(100%)*	0	0
IAA-B	0	0	0	0
CAT	0	0	0	0
Other ectopic vessels	15 *(45*.*5%)*	2 *(40%)*	0	0
RAA	7 *(21*.*2%)*	0	0	0
Over-riding Aorta	9 *(27*.*3%)*	1 (20%)	0	0
DORV	8 *(24*.*2%)*	0	0	0
mVSD	33 *(100% incl*. *DORVs and OA))*	5 *(100%)*	1 *(3*.*0%)*	1 *(3*.*8%)*

CAT, common arterial trunk; DORV, double outlet right ventricle; IAA-B, interrupted aortic arch type B; LCC, left common carotid; LSA, left subclavian; OA, over-riding aorta; RAA, right-sided aortic arch; RSA, right subclavian; mVSD, membranous ventricular septal defect.

**Fig 2:**
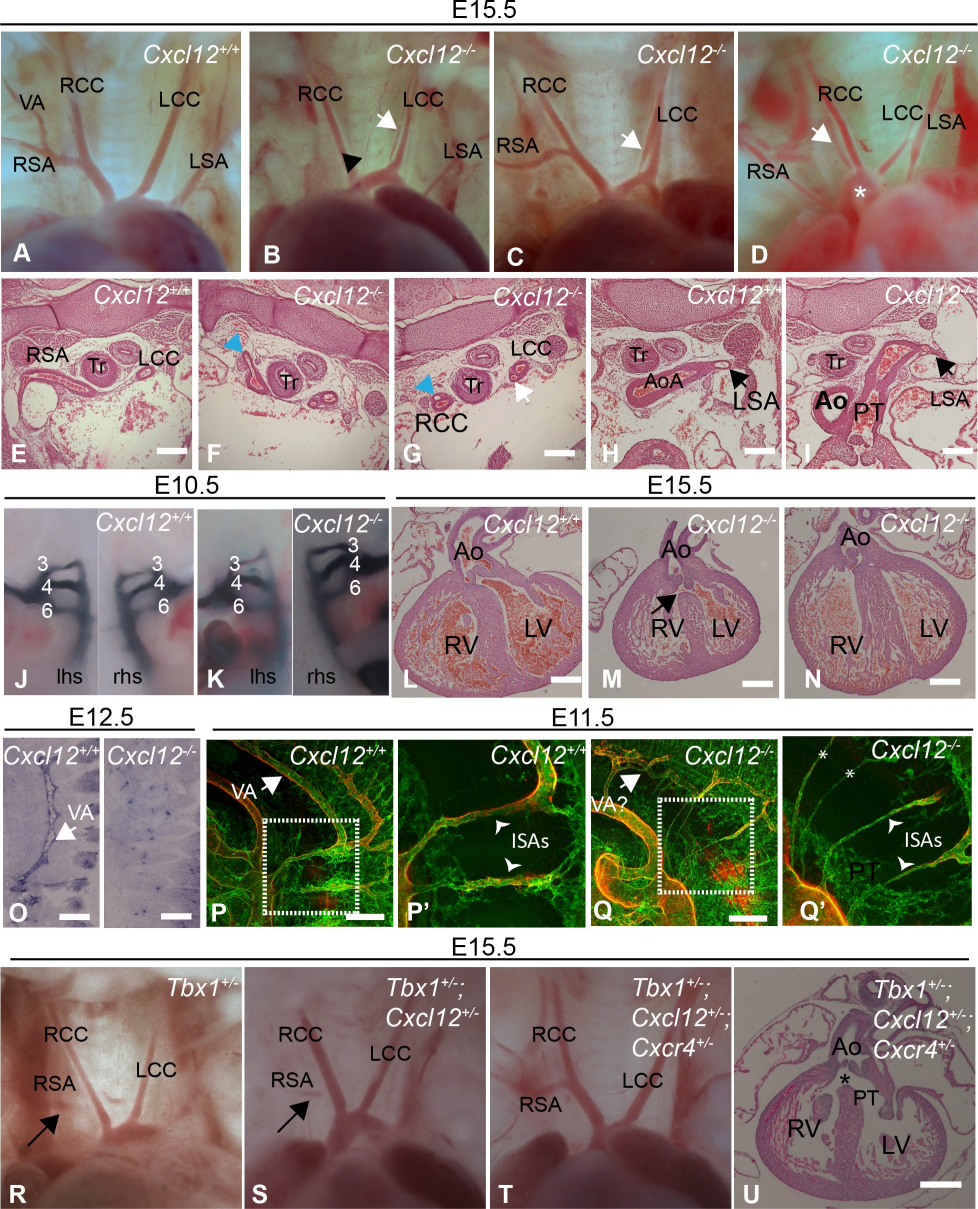
Mutation of *Cxcl12* causes cardiovascular defects but does not synergise with Tbx1 heterozygosity. **(A-I)** Aortic arch anomalies in *Cxcl12* mutants at E15.5 including duplicated LCC (white arrows in B, C and G), ectopic vessels originating from the aortic arch (black arrowhead in panel B), right-sided aortic arch (RAA, Panel D) and RSA apparently absent (B)- however note thin vessel branching off and running parallel with and dorsal to the RCC (blue arrowheads in F and G, serial sections)- or unbranched (C). The LSA was thin and abnormally positioned (panel B) or not visible (C), originating from a more distal part of the aorta in *Cxcl12*^*-/-*^embryos compared to controls (black arrows in panels H and I). **(J, K)** India ink injections of E10.5 embryos showed PAA3-6 were normal in both *Cxcl12* mutants (n = 8) and controls (n = 9). **(L-N)** Outflow alignment defects in *Cxcl12* mutants at E15.5 e.g. VSD and over-riding aorta (arrow in panel M), and DORV (panel N shows aorta opening into the right ventricle. **(O-Q)** Development of the intersomitic arteries (ISAs) and vertebral artery (VA) is affected in *Cxcl12* mutants. *In situ* hybridisations (sagittal sections) show expression of *Cxcr4* in the VA at E12.5 and absence of the VA in *Cxcl12* ^-/-^ embryos (O). Confocal analysis of wholemount PECAM (green) and SM22α-stained (red) E11.5 embryos shows malformation of the VA (arrows) in the *Cxcl12* mutant (Q,) compared to control (P) and hyperplasia of ISAs (indicated by arrowheads; boxed regions are shown enlarged in P’ and Q’- z projections created from shorter confocal substacks show the ISAs more clearly), and failure to regress of some anterior ISAs (asterisks). **(R-U)** *Tbx1* and *Cxcl12/Cxcr4* do not synergise in producing aortic arch phenotypes. Retro-oesophageal RSA was observed in a minority of mutants with a *Tbx1* mutant allele (e.g. R and S), whilst the majority of *Tbx*^*+/-*^*;Cxcl12*^*+/-*^ and *Tbx*^*+/-*^*;Cxcl12*^*+/-*^*;Cxcr4*^*+/-*^ mutants showed normal vessel patterning (T). mVSDs were observed at low frequency in double and triple heterozygotes (U). Ao, aorta; AoA, aortic arch; DORV, double outlet right ventricle; LCC, left common carotid; LSA, left subclavian; LV, left ventricle; mVSD, membraneous ventricular septal defect; PAA, pharyngeal arch arteries;PT, pulmonary trunk; RCC, right common carotid; RSA, right subclavian; RV, right ventricle; Tr, trachea. Scale bars represent 200μ in panels in E-I and O, and 500μ in panels L-N and P-Q.

In summary, while all *Cxcl12* and *Cxcr4* (not shown) constitutively null embryos had a membranous VSD at E15.5 we found no examples of CAT, IAA-B or retro-oesophageal RSA, nor did we see any examples of pulmonary atresia (part of the tetralogy of Fallot spectrum). However, all *Cxcl12* and *Cxcr4* null mutant embryos had defects of the subclavian arteries on at least one side, and both sides were affected in the majority of mutants. Importantly, these defects were distinct from the defects observed in *Tbx1* mutants. On the right side, the subclavian artery was frequently not visible until sectioned (Fig 2B, blue arrowhead in 2F and 2G), or where visible lacked arterial branches including the vertebral artery (Fig 2C). On the left side the subclavian was generally positioned abnormally distally and also lacked arterial branches (Fig 2B, 2C and 2I). Other vessel defects not reported in *Tbx1* mutants were also detected, predominantly ectopic vessels that appeared as duplicated carotid arteries (arrows in Fig 2B, 2C and 2G) or vessels arising from other parts of the aortic arch (arrowhead in Fig 2B). In the more distal part of the LCCs duplication was only evident upon sectioning, which showed two lumen existing within the same structure (Fig 2G). However, ink injections at E10.5 showed normal formation of the PAAs in *Cxcl12* mutants (Fig 2J and 2K). Right-sided arch and alignment defects such as over-riding aorta and double outlet right ventricle (DORV) were also found in *Cxcl12* mutants (Fig 2D, 2M and 2N).

*In situ* hybridisations in E12.5 embryos showed expression of *Cxcr4* in the vertebral artery, a branch of the subclavian artery, and confirmed the absence of this vessel in *Cxcl12* mutants (Fig 2O). As the distal portion of the right subclavian (RSA) and the entirety of the left subclavian (LSA) are derived from the seventh intersomitic arteries (proximal portions of the RSA are derived from the right 4^th^ PAA and the right dorsal aorta) [25, 26], we wanted to analyse the intersomitic arteries in *Cxcl12* mutants at E11.5 to see if defective development of these vessels could explain the mutant phenotype. Confocal analysis revealed thinner vessels with fewer anastomoses in the *Cxcl12* nulls (Fig 2P–2Q’). Malformation of the vertebral artery was also observed, along with failure of some anterior intersomitic arteries to regress (Fig 2Q and 2Q’). CXCR4 expression was apparent in the developing intersomitic arteries from E9.5 onwards, (S1A–S1F Fig), with strong expression of *Cxcl12* in the surrounding mesenchymal tissue at E11.5 (S1G and S1H Fig, serial sections). However, the intersomitic arteries appeared normal in *Cxcl12* mutants at E9.5–10. 5 (not shown) showing that CXCL12/CXCR4 signalling is not required for the formation or early development of these vessels.

While IAA-B, retro-oesophageal RSA and CAT were not observed following loss of *Cxcl12* or *Cxcr4* we reasoned that it was still possible that diminished CXCL12-CXCR4 signalling might interact with TBX1-dependent developmental pathways. We therefore examined *Tbx1*^*+/-*^*;Cxcl12*^*+/-*^ double heterozygote embryos for evidence of synergistic effect of these alleles upon aortic arch phenotypes (Table 2, Fig 2R and 2S). No significant interaction was observed. In order to impact the signalling pathway further we examined *Tbx1*^*+/-*^*;Cxcl12*^*+/-*^*;Cxcr4*^*+/-*^ embryos (Table 2, Fig 2T and 2U). Simplistically, if there is half the amount of ligand and half the amount of receptor to interpret the signal the pathway would be at 25% activation level, less if *Tbx1* haploinsufficiency impacts the pathway, although we have no means of precisely quantitating whether this is the case. No significant effect on phenotype was observed, and no right-sided arches were observed.

**Table 2:** Cardiac anomalies in *Tbx1* and *Cxcl12* pathway double and triple heterozygotes at E15.5t.

Defect	Tbx^+/-^(n = 27)	Tbx1^+/-^(previously published, n = 61)^a^	Tbx1^+/^; Cxcl12^+/-^(n = 26)	Tbx1^+/-^; Cxcl12^+/^;Cxcr4^+/-^(n = 13)
Retro RSA	5 *(18*.*5%)*	9	4 *(15*.*4%)*	3 *(23*.*0%)*
High take-off RSA	0	0	1 *(3*.*8%)*	0
IAA-B	2 *(7*.*4%)*	4	0	0
RAA	0	Not scored	0	0
mVSD	0^b^	Not scored	1^c^ *(6*.*7%)*	2^d^ *(15*.*4%)*

^a^ Data from previously published studies examining vessel defects in *Tbx1* mutant embryos on the same background, at E15.5 [5, 24] and E14.5 [22].

^b^ 4 hearts sectioned

^c^ 15 hearts sectioned

^d^ 11 hearts sectioned, 2 analysed by OPT

IAA-B, interrupted aortic arch type B; RAA, right-sided aortic arch; RSA, right subclavian; mVSD, membranous ventricular septal defect.

### Lineage specific requirements for CXCL12-CXCR4 signalling

**Fig 3:**
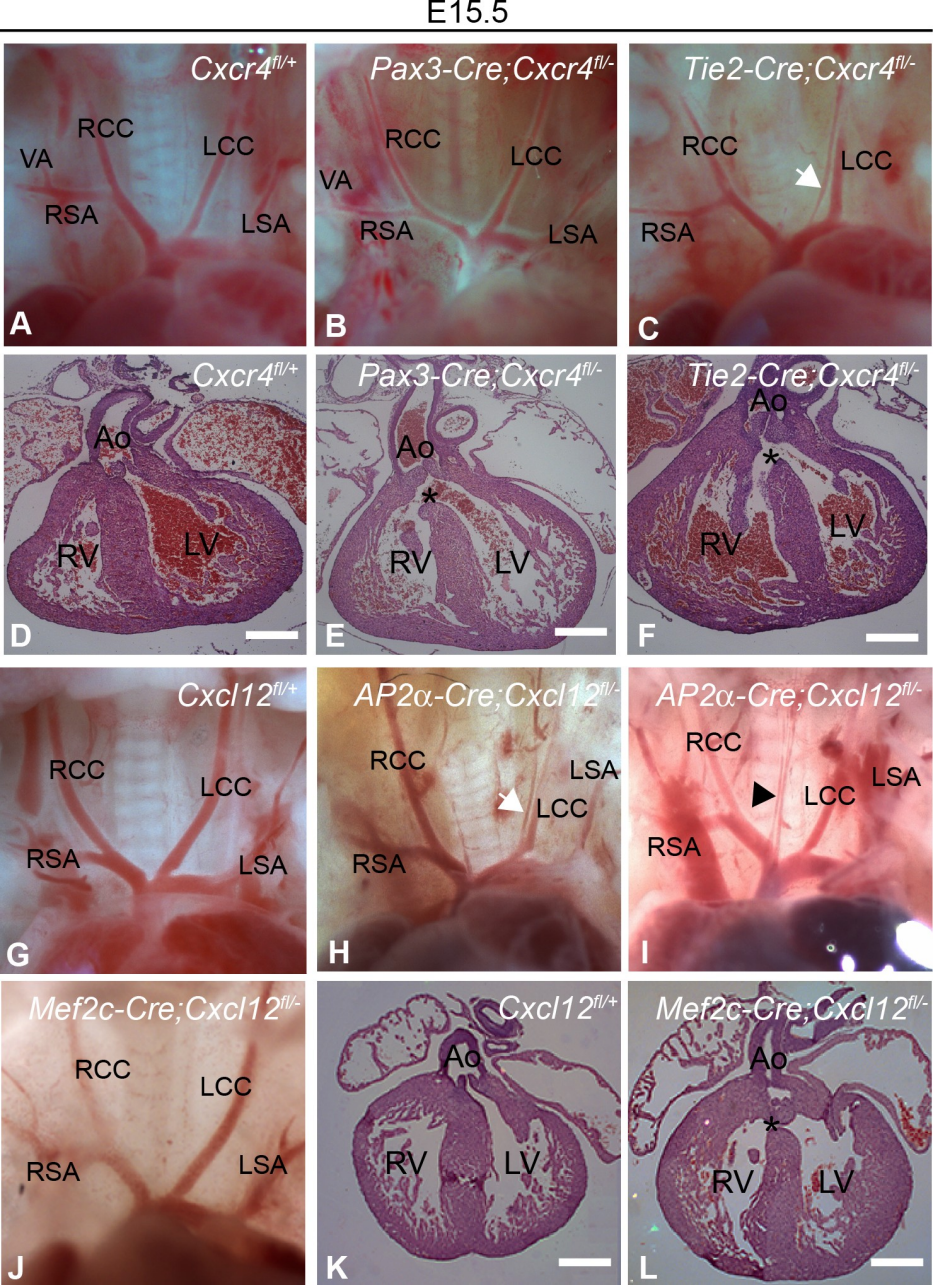
Cardiovascular defects in *Cxcr4* and *Cxcl12* conditional mutants. **(A-F)** Knock-out of *Cxcr4* in NCC and endothelial lineages. Aortic arches and vasculature were normal in *Pax3-Cre;Cxcr4*^*fl/-*^ mutants (B) but mVSDs were present in 23.5% of embryos examined (asterisk in E indicates VSD with over-riding aorta). *Tie2-Cre; Cxcr4*^*fl/-*^ mice phenocopy both aortic arch and intracardiac defects (C, F) e.g. unbranched RSA, absent or mis-positioned LSA, and an ectopic vessel arising from the aortic arch (arrow in C); asterisk (F) indicates VSD with over-riding aorta. **(G-L)** Knock-out of *Cxcl12* in ectoderm and second heart field. *Ap2*α*-Cre;Cxcl12*^*fl/-*^ mutants displayed a subset of aortic arch defects only i.e. duplicated LCC in a single case (arrow, H) and ectopic vessels arising from the aortic arch in approximately 40% of embryos examined (arrowhead, I). Aortic arches and vasculature were normal in *AHF-Mef2cCre;Cxcl12*^*fl/-*^ embryos (J) whereas membranous VSDs were found in 36.4% of embryos examined (arrow in L). Ao, aorta; AoA, aortic arch; DORV, double outlet right ventricle; LCC, left common carotid; LSA, left subclavian; LV, left ventricle; mVSD, membranous ventricular septal defect; Oe, oesophagus; PT, pulmonary trunk; RCC, right common carotid; RSA, right subclavian; RV, right ventricle; Tr, trachea; VA, vertebral artery. Scale bars represent 500μ.

These data suggest that absence of *Cxcl12* or *Cxcr4* is in itself insufficient to cause defects typical of *Tbx1* haploinsufficiency. To support this conclusion and to test whether absence of CXCR4 in NCC contributes to abnormal subclavian artery morphogenesis or other cardiovascular defect(s), we undertook conditional mutagenesis using the well characterised *Wnt1Cre* driver. Of six *Wnt1Cre;Cxcr4*^*fl/fl*^ embryos examined at E15.5, none had great vessel defects, head/neck vessel defects or VSDs (Table 3). It has been noted that *Pax3Cre* may be active in a slightly earlier NCC population and can give a phenotype where a *Wnt1Cre* driver does not [27, 28]. Of 17 *Pax3Cre;Cxcr4*^*fl/-*^ embryos again none had aortic arch defects, although 4 had a membranous VSD (Fig 3B and 3E). No outflow tract defects were observed (Table 3). No vessel defects or VSDs were found in 8 *Pax3-Cre*^*+/-*^ controls. Interpretation of the presence of VSDs is somewhat complicated by possible interaction between *Pax3* and *Cxcr4*: *Pax3* has a known haploinsufficiency in the NCC and the *Cre* used is a knock in allele [29–31]. In contrast, deletion of *Cxcr4* from the endothelial lineage with *Tie2-Cre* yielded frequent abnormalities of the carotid and/or subclavians (Table 3 and Fig 3C), with defects found in 76% of conditional mutants (n = 21) compared with 18 *Tie2-Cre;Cxcr4*^*fl*/*+*^ controls which were all normal. Membranous VSDs (including one over-riding aorta) were found in 57% of *Tie2-Cre* conditionals examined (n = 14) (Table 3, Fig 3F). Thus, allowing for reduced penetrance using this *Cre* driver [17], loss of *Cxcr4* from endothelial cells substantially recapitulated the constitutively null phenotype.

**Table 3:** Cardiovascular anomalies in conditional mutants.

***Cxcr4***			
**Cre driver**	**Tissue targeted**	**Aortic arch defects**	**VSD**
Wnt1 (n = 6)	Neural crest	0	0
Pax3 (n = 17)	Neural Crest	0	4 *(23*.*5%)*
Tie2 (n = 21^a^)	Endothelium	16 *(76%)* aberrant RSA:12 aberrant LSA:8 duplicate LCC:7	8 *(57%)* (n = 14)
***Cxcl12***			
**AP2α** (n = 12^b^)	Ectoderm/neural crest	5 *(41*.*7%)* duplicate LCC:1 ectopic vessels: 5	0
Mef2c (n = 11)	Second heart field	0	4 *(36*.*4%)*

^**a**^ Of the 21 total *Tie-Cre/Cxcr4* conditional mutants analysed for vessel defects 8 were *Tie2-Cre;Cxcr4*^*fl/fl*^ and 13 were *Tie2-Cre;Cxcr4*^*fl/-*^

^**b**^ 5 embryos were sectioned and 7 were analysed by OPT

LCC, left common carotid; LSA, left subclavian; RSA, right subclavian

We next sought to identify which source(s) of CXCL12 was (were) required for mediating the effects seen following loss of *Cxcr4* from endothelial cells. We used *Ap2*α*-Cre* [32] to target *Cxcl12* in the pharyngeal surface ectoderm (this transgene is also active in the NCC, however a recent study reported an entirely normal cardiovascular system following *Wnt1Cre* ablation of *Cxcl12* [33]). 12 *Ap2*α*-Cre;Cxcl12*^*fl/fl*^ embryos were examined and 5 showed vessel defects, albeit considerably milder than observed in constitutive *Cxcl12* nulls. However, small ectopic vessels emerging from the aorta were present (Table 3, Fig 3H and 3I). None of these embryos had a VSD. No defects were found in a total of 11 littermate control embryos (*Ap2*α*-Cre;Cxcl12*^*fl/+*^ *and Cxcl12*^*fl/+*^) examined concurrently. Conversely, of 11 *AHFMef2cCre;Cxcl12*^*fl/fl*^ mutants, 4 had a membranous VSD, but none had a vessel defect (Table 3, Fig 3J and 3L); this Cre is active in the second heart field [34]. Littermate controls *AHFMef2c-Cre;Cxcl12*^*fl/+*^ *(n = 6) and Cxcl12*^*fl/+*^ *(n = 6*) had no defects. Thus, CXCL12 is required in pharyngeal surface ectoderm for aspects of normal neck vessel development and the second heart field for membranous interventricular septation.

## Discussion

TBX1 has cell autonomous and non-cell autonomous effects that are essential for normal cardiovascular morphogenesis. Here we addressed the recent hypothesis that a non-autonomous effect on cardiac NCCs via the ligand CXCL12 acting through the receptor CXCR4 has a role in this regard. Certainly, results from other model organisms demonstrate a role for CXCL12-CXCR4 signalling in NCC migration. Of most relevance is work in the chick where dominant negative inhibition of CXCR4 activity in cardiac NCC gave rise to cardiac defects (although no arch artery or aortic arch analyses were specifically mentioned) [35]. Following up on previous work, we established that *Cxcl12* expression in pharyngeal surface ectoderm and rostral mesoderm is reduced in the absence of *Tbx1*. We then asked three main questions with regard to the potential role of the CXCL12:CXCR4 pathway in 22q11DS: to what extent do the defects in CXCL12 signalling mutants recapitulate *Tbx1* mutation? Is CXCL12 signalling from the pharyngeal surface ectoderm to the cardiac NCC responsible for heart and vessel defects seen in *Cxcl12* mutants (and, if not, what source and target lineages are involved)? And finally, does reduction in CXCL12-mediated signalling exacerbate *Tbx1* haploinsufficiency?

We showed that, in concordance with a recent study [33], while homozygous mutation of either *Cxcl12* or *Cxcr4* yielded intracardiac defects, we found neither retro-oesophageal RSA, IAA-B, nor CAT (fully penetrant in *Tbx1* nulls) in these mice. Previous work has identified that *Tbx1* expression in pharyngeal surface ectoderm is required for normal NCC patterning and formation of the fourth pharyngeal arch arteries. However, loss of CXCL12 from the pharyngeal surface ectoderm produced only minor ectopic vessel phenotypes in the mutant embryos, whilst formation of PAAs including the 4^th^ PAA was normal, and no intracardiac defects were found. Loss of CXCL12 from the *AHFMef2c* lineage was sufficient to cause intracardiac defects. We therefore conclude that CXCL12 from two (at least) independent sources is required for cardiovascular morphogenesis. The duplicated carotid phenotype and right-sided arch were not extensively recapitulated in these experiments; it may be that the recombination of the conditional allele was insufficiently efficient, or that CXCL12 from remaining source lineage(s) is sufficient to prevent these abnormalities arising. In addition, the Cre drivers used would not be expected to target expression of *Cxcl12* in the mesenchyme surrounding the ISAs, accounting for the lack of subclavian defects in the conditional mutants. Of interest, *Nkx2*.*5Cre;Cxcr4*^*fl/-*^ mutants survived normally through adulthood with no apparent deficiencies [36]. As this driver is active in the first and, to a lesser extent, second heart fields, targeting atrial and ventricular endocardium and myocardium, but not most vascular endothelium, this is consistent with our interpretation that multi-source CXCL12 signalling to CXCR4- expressing rostral endothelial cells is required for cardiovascular development.

Murine *Tbx1* heterozygosity has been shown to interact with heterozygosity of a number of other genes with a resultant increase in the penetrance and or severity of defects e.g. *Fgf8* [6], *Wnt5a* [37], *Smad7* [24], *Gbx2* [5], and *Chd7* [22]. We were unable to detect any exacerbation of the *Tbx1*^*+/-*^ phenotype on a background of *Cxcl12* heterozygosity, with or without concomitant heterozygosity for *Cxcr4*. While a larger study might have uncovered a small effect the triple heterozygote study in particular suggests that down-regulation of the CXCL12-CXCR4 pathway does not have a major role in mediating *Tbx1*^*+/-*^ haploinsufficient phenotypes.

Taken together, our data are consistent with a role for CXCL12 signalling from pharyngeal surface ectoderm and mesenchyme to endothelial cells and NCCs via CXCR4, with endothelial cells being the more important cell type in the context of cardiovascular morphogenesis. This is consistent with a recent report that demonstrated a requirement for CXCL12 signalling to CXCR4 positive endothelial cells during development of the lung vasculature (not examined in our study) as well as neck vessel development [33]. Both this study and our own work detected subclavian artery and vertebral artery defects in *Cxcl12* null embryos. These arteries have contributions from remodelling ISAs. While ISAs were normal at E9.5, from around E11 onwards we observed thinner intersomitic arteries with fewer anastomoses in *Cxcl12* nulls. Thus, the phenotypes we observe in neck vessels are likely to be associated with defective remodelling of the intersomitic arteries rather than PAAs.

There are differences between mouse and chick which might underlie the distinct loss of function phenotypes. In chick, CXCR4 down-regulation by *miRNA* led to NCC apoptosis which could explain the defects seen in that organism [15]. As right-sided arch was observed in approximately 20% of *Cxcl12* null embryos, but in not double or triple heterozygotes; it is feasible that chick is more sensitive than mouse to down-regulation of CXCL12. Speculatively, CXCR7 (or another receptor) could compensate in mouse, but not chick. However, such compensation would not be possible in the *Cxcl12* constitutive nulls as ligand would be entirely lacking. More generally, it is now accepted that for several genes mouse knockouts fail to show phenotypes analogous to those seen in fish, frog or chick manipulations [38]. Whether humans are closer to mice or non-mammals is of course untested, but it seems likely the two mammals are more closely related from the developmental perspective. Finally, from the point of view of defective aortic arch morphogenesis in *Tbx1* heterozygotes, we note that the relevant signalling mechanism(s) originating in pharyngeal surface ectoderm, downstream of TBX1 and communicated to NCCs remains to be discovered.

## Materials and methods

### Mice and animal husbandry

Mice were housed and bred according to UK Home Office regulations. *Cxcl12* mutants *Cxcl12-GFP* [39], and *Cxcl12* ^*fl/fl*^ [40], *Cxcr4* ^*fl/fl*^ [41], *Ap2*α*-Cre* [32], *Pax3-Cre* [29], *Tbx1* [42] *Tie2-Cre* [43], *AHFMef2c-Cre* [34] and *Wnt1-Cre* mice [44] have been described previously.

*Cxcr4* null mice were obtained by crossing *Cxcr4* ^*fl/fl*^ mice with βactin‐Cre mice [45]; offspring carrying both *Cre* and recombined *Cxcr4* alleles were bred again with wild types to obtain offspring carrying only the recombined *Cxcr4* allele (*Cxcr4*^*+/-*^). *Tie2‐Cre*;*Cxcr4*^*fl/-*^ embryos were generated by crossing *Tie2‐Cre;Cxcr4*^*+/-*^ with *Cxcr4* ^*fl/fl*^ mice^*-*^; *Tie2‐Cre*;*Cxcr4*^*fl/fl*^ embryos were generated by crossing *Tie2‐Cre; Cxcr4*^*fl/+*^ with *Cxcr4* ^*fl/fl*^ mice. Similarly, *Pax3‐Cre; Cxcr4*^*fl/-*^ embryos were generated by crossing *Pax3‐Cre;Cxcr4*^*+/-*^ with *Cxcr4* ^*fl/fl*^ mice and *Wnt1-Cre*;*Cxcr4*^*fl/fl*^ by crossing *Wnt1-Cre*;*Cxcr4*^*fl/+*^ with *Cxcr4* ^*fl/fl*^ mice. *Ap2*α*-Cre* and *Mef2c‐Cre Cxcl12* conditional embryos were bred by crossing *Cxcl12* ^*fl/fl*^ mice with *Ap2*α*-Cre*; *Cxcl12*^*+/-*^ or *Mef2c‐Cre*; *Cxcl12*^*+/-*^ mice respectively. Pax3-Cre mice were crossed with ROSA26R^eYFP/eYFP^ mice [46] in order to lineage-label neural crest in the resulting embryos. For timed pregnancies the date of the vaginal plug was designated E (embryonic age) 0.5. All mice were maintained on a C57Bl/6 background.

In this study animal sacrifice was carried out by exposure to rising levels of carbon dioxide followed by cervical dislocation, or by cervical dislocation followed by decapitation. Animal work was carried out according to UK Home Office regulations under project license number PPL 70 6875 2518. The project licence application was approved by the UCL Animal Welfare and Ethical Review Body (AWERB) before submission to the Home Office.

### Histology

Embryonic hearts were dehydrated through an ethanol series, embedded in paraffin wax, sectioned and stained with haematoxylin and eosin using standard methods.

### Confocal analysis

Confocal analysis was performed as described previously [17, 47]. Immunolabelled embryos (E9.5–11.5) were dehydrated through a methanol series before clearing with 1 part Benzyl alcohol/ 2 parts benzyl benzoate solution (BABB, Sigma) and examined by epifluorescence on an inverted LSM710 confocal system mounted on an AxioObserver Z1 microscope (Carl Zeiss Ltd, United Kingdom) [17, 47].

### Optical projection tomography

OPT was carried out as previously described [48]. Briefly, E15.5 embryos were dissected out, and after chilling for at least 1–2 hours at 4°C, the trunks were opened and cartilage from the rib cage was removed. They were then fixed overnight in 4% PFA/PBS and mounted in low-melting agarose (Life Technologies). Samples were trimmed to remove excess agarose and washed in 100% methanol followed by clearing in BABB. Scanning was undertaken using a Bioptonics OPT Scanner 3001M (MRC Technology, Edinburgh, UK). NRecon software (Skyscan NV) was used for image reconstruction from projections using a back-projection algorithm.

### Ink injections

Ink injection was performed on E10.5 embryos fixed in 4% paraformaldehyde overnight at 4°C by targeting the outflow tact with a microinjection needle filled with Indian ink.

### *In situ* hybridisation

*In situ* hybridisation was carried out on wholemount embryos or 10μm paraffin sections based on standard methods [49] and using digoxygenin-labelled probes for *Cxcl12*, *Cxcr4* and *Sox10* as described previously [5, 17]. Paraffin sections were de‐waxed (Histoclear, National Diagnostics) and re‐hydrated through an ethanol series. Sections were permeabilised using Proteinase K (Sigma) at 20μg/ml for 8 min. This was followed by glycine at 2mg/ml for 5 min, washing in PBS and post‐fixing in 4% paraformaldehyde for 20 min. After rinsing in PBS, sections were pre‐hybridised in hybridisation buffer (50% de‐ionised formamide (Promega), 5xSSC pH 5.0, 50μg/ml yeast tRNA (Sigma), 1% SDS and 50μg/ml heparin (Sigma) for one hour at 70°C. Probe incubation was carried out at 70°C overnight followed by washes at 65°C as follows: 3x 15 min in 50% formamide/5x SSC/1% SDS and 2 x 15 min in 50% formamide/2x SSC. The samples were allowed to cool to room temperature then washed twice for 10 min each in MABT (100mM maleic acid, 150mM NaCl, 0.1% Tween‐20, pH 7.5) with 2mM tetramisole hydrochloride (Levamisol, Sigma). Blocking was carried out in 2% Blocking Reagent (Roche Life Science)/5% sheep serum/5% goat serum (in MABT) for one hour at room temperature before incubating with anti‐Digoxygenin‐AP antibody at 1:2000 (4°C overnight). After removal of the antibody the sections were washed in MABT/levamisol and equilibrated in alkaline phosphatase buffer (2 x 5 min washes) before staining with BM Purple solution (Roche Life Science).

### Immunolabelling

Immunolabelling of 10μ frozen sections (in OCT) was carried out according to standard protocols. Briefly, sections were permeabilised in 0.5% Triton X-100 (Sigma) for 5 minutes, rinsed in PBS twice, then incubated in blocking buffer for one hour at room temperature (PBS/10% BSA/10% goat serum/0.1% Triton X-100) followed by primary antibody in blocking buffer at 4°C overnight. After 3x 5 minute washes in PBS, secondary antibodies in blocking buffer were applied for one hour at room temperature; DNA was counterstained using DAPI (Sigma). Fluorescent images were captured on a Zeiss Axio Lumar.V12 stereomicroscope.

For whole-mount immunolabelling, hearts were permeabilised in PBST (PBS/0.1% Tween-20), blocked for one hour in PBST/10% goat serum, and incubated overnight at 4°C with primary antibody in blocking buffer. Hearts were washed several times in PBST (one hour washes) before incubating overnight at 4°C with secondary antibody. After several PBST washes, the immunolabelled embryos were dehydrated through a methanol series before clearing with BABB.

### Antibodies

#### Primary antibodies

Anti‐CXCR4 (rabbit polyclonal UMB2, Abcam) used at 1:300, anti‐PECAM-1 (Armenian hamster monoclonal 2H8, Thermo Scientific Pierce) at 1:400, anti-GFP (chicken polyclonal, Abcam), used at 1:500, Ap2α (3B5, Santa Cruz Biotechnology) used at 1:25, SM22α (rabbit polyclonal, Abcam) used at 1:250.

#### Secondary antibodies

Goat anti-Armenian hamster Alexa Fluor 488 (Abcam), goat anti-Chicken Alexa Fluor 488, goat anti-Mouse Alexa Fluor 594, goat anti-Rabbit Alexa Fluor 647 and 594 (Life Technologies). All secondary antibodies were diluted 1:500.

## Supporting information

S1 Fig Expression of Cxcr4 and Cxcl12 in the developing intersomitic arteries (ISAs). **(A-F)** Immuno-labelling of ISAs (arrows) with PECAM (green) and CXCR4 (red) antibodies, in sagittal sections of wild type embryos at E9.5 (A-C) and E11.5 (D-F). **(G, H)** *In* situ hybridisations show expression of *Cxcr4* and *Cxcl12* in serial sagittal sections of E11.5 wild type embryos. ISAs are indicated by arrows. Note strong expression of *Cxcl12* in the mesenchyme surrounding the ISAs. Scale bars represent 100μ in panels A-F and 200μ in panels G and H.
(TIF)
