# Supplementary figures and images for: Loss of CXCL12/CXCR4 signalling impacts several aspects of cardiovascular development but does not exacerbate Tbx1 haploinsufficiency
Source: PLoS One. 2018 Nov 8;13(11):e0207251. doi: 10.1371/journal.pone.0207251 (PMC6224166; doi:10.1371/journal.pone.0207251)

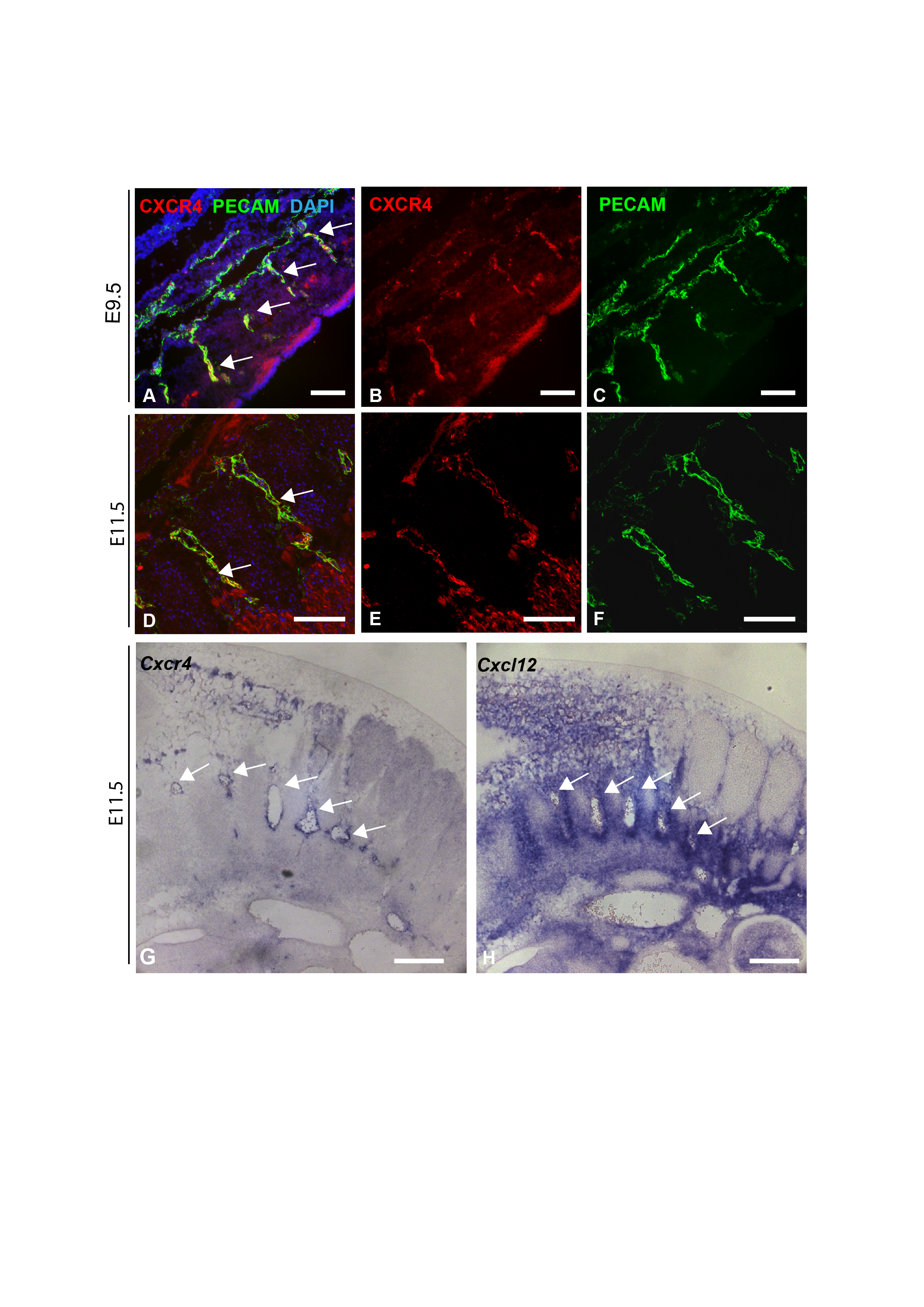

Supplement: S1 Fig — (A-F) Immuno-labelling of ISAs (arrows) with PECAM (green) and CXCR4 (red) antibodies, in sagittal sections of wild type embryos at E9.5 (A-C) and E11.5 (D-F). (G, H) In situ hybridisations show expression of Cxcr4 and Cxcl12 in serial sagittal sections of E11.5 wild type embryos. ISAs are indicated by arrows. Note strong expression of Cxcl12 in the mesenchyme surrounding the ISAs. Scale bars represent 100μ in panels A-F and 200μ in panels G and H. (TIF) [file pone.0207251.s001.tif]
